# Folic acid-mediated hollow $\text {Mn}_{3}\text {O}_{4}$ nanocomposites for in vivo MRI/FLI monitoring the metastasis of gastric cancer

**DOI:** 10.1186/s12938-024-01248-6

**Published:** 2024-06-10

**Authors:** Zhihua Yang, Chenying Wang, Shangting Du, Qin Ma, Wei Wang, Changhu liu, Yonghua Zhan, Wenhua Zhan

**Affiliations:** 1 Department of Radiation Oncology, General Hospital of Ningxia Medical University, Yinchuan, 750004 Ningxia China; 2 School of Life Science and Technology, Xidian University and Engineering Research Center of Molecular and Neuro Imaging, Ministry of Education, Xi’an, 710126 Shaanxi China

**Keywords:** Metastasis, $\text {Mn}_{3}\text {O}_{4}$, Nanocomposite, Magnetic resonance imaging, Fluorescence imaging, Gastric cancer

## Abstract

**Background:**

Metastasis is one of the main factors leading to the high mortality rate of gastric cancer. The current monitoring methods are not able to accurately monitor gastric cancer metastasis.

**Methods:**

In this paper, we constructed a new type of hollow $\text {Mn}_{3}\text {O}_{4}$ nanocomposites, $\text {Mn}_{3}\text {O}_{4}$@HMSN-Cy7.5-FA, which had a size distribution of approximately 100 nm and showed good stability in different liquid environments. The in vitro magnetic resonance imaging (MRI) results show that the nanocomposite has good response effects to the acidic microenvironment of tumors. The acidic environment can significantly enhance the contrast of ${\text {T}_{1}}$-weighted MRI. The cellular uptake and endocytosis results show that the nanocomposite has good targeting capabilities and exhibits good biosafety, both in vivo and in vitro. In a gastric cancer nude mouse orthotopic metastatic tumor model, with bioluminescence imaging’s tumor location information, we realized in vivo MRI/fluorescence imaging (FLI) guided precise monitoring of the gastric cancer orthotopic and metastatic tumors with this nanocomposite.

**Results:**

This report demonstrates that $\text {Mn}_{3}\text {O}_{4}$@HMSN-Cy7.5-FA nanocomposites is a promising nano-diagnostic platform for the precision diagnosis and therapy of gastric cancer metastasis in the future.

**Conclusions:**

In vivo MRI/FLI imaging results show that the nanocomposites can achieve accurate monitoring of gastric cancer tumors in situ and metastases. BLI’s tumor location information further supports the good accuracy of MRI/FLI dual-modality imaging. The above results show that the MHCF NPs can serve as a good nano-diagnostic platform for precise in vivo monitoring of tumor metastasis. This nanocomposite provides more possibilities for the diagnosis and therapy of gastric cancer metastases.

## Introduction

**Fig. 1:**
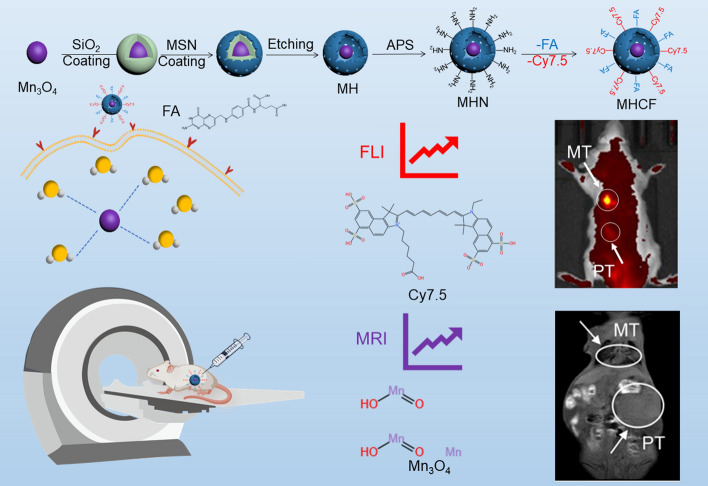
The mechanism of hollow $\text {Mn}_{3}\text {O}_{4}$ nanocomposites for in vivo MRI/FLI guided monitoring the metastasis of gastric cancers

Gastric cancer is a common and highly fatal malignant tumor of the digestive system. The incidence of gastric cancer is relatively high worldwide, ranking fourth in terms of mortality among all malignant tumors [1, 2]. Since their early symptoms are relatively insidious, gastric cancer are usually diagnosed at the advanced stage, at which time the lymph node metastasis, blood metastasis, and distant organ metastasis are very likely to occur [3]. Regardless of whether gastric cancer has metastasis to a single organ or multiple organs, the treatment will be difficult [4]. Therefore, accurate monitoring of gastric cancer metastasis will help us treat gastric cancer in a timely manner to prevent poor prognosis [5, 6]. However, effective monitoring methods for gastric cancer metastasis are limited. Tissue biopsy is an invasive pathological diagnosis method [7]. The type and progression of gastric cancer can be further determined by observing and analyzing tissue sections. However, tissue biopsy is time sensitive and cannot detect potential and undetected metastasis. Additionally, gastric cancer metastasis can also be detected by circulating tumor cells (CTC) in body fluids. Qi et al. predicted the risk of gastric cancer metastasis by detecting CTC cDNA [8, 9]. However, there are always certain discrepancies between the indicators of CTC and the actual metastasis samples, and the specific location of metastatic lesions cannot be determined by CTC. Therefore, establishing an effective method for accurate detection of gastric cancer metastasis has become a top priority in clinical diagnosis and treatment. As a non-invasive in vivo monitoring method, imaging has been widely used in the monitoring of gastric cancer in recent years [6]. Qin et al. constructed $^{68}\text {Ga}$-DOTA-FAPI-04 ($^{68}\text {Ga}$-FAPI), which can better display gastric cancer in situ tumors and metastatic lesions compared with the traditional fluorodeoxyglucose ($^{18}\text {F}$-FDG) PET/CT imaging. However, due to the low sensitivity of $^{18}\text {F}$-FDG PET/CT for gastric cancer, false-positive results may occur due to excessive uptake of $^{18}\text {F}$-FDG by the stomach wall, and distant lymph node metastasis may also be missed. Therefore, Wang et al. applied $^{68}\text {Ga}$-FAPI PET/MR to monitor tumor metastasis and found that MR could help further monitor the primary tumor, lymph node metastasis, and peritoneal metastasis [7–9]. However, although PET imaging has high sensitivity, its large-scale application in clinic is restricted due to its high radiation, low resolution, and high price. On the other hand, magnetic resonance imaging (MRI) has been increasingly applied in gastric cancer monitoring because of its relatively high resolution of soft tissue and non-radiation [10, 11]. However, MRI has low sensitivity and cannot perform whole-body imaging. Therefore, it is very important to establish an MRI-based multi-modal imaging method for accurate monitoring of gastric cancer metastasis. Fluorescence imaging (FLI), as an emerging high-sensitivity imaging technology, can effectively visualize tumor lesions [11]. Its combination with MRI has been successfully used for accurate and effective monitoring of tumors in vivo. Li et al. developed a PEG-Cy-Fs that has the imaging capabilities of F-19 MRI and near-infrared fluorescence imaging (NIR FLI) for the diagnosis and treatment of mouse breast cancer [12]. Chen et al. designed a Gd-PPNs nanoparticle with ${\text {Gd}^{3+}}$ as a ${\text {T}_{1}}$-weighted MRI contrast agent and tetrakis (4-carboxyphenyl) porphyrin (TCPP) as a fluorescence imaging organic medium. Gd-PPNs had significant effects in imaging pancreatic cancer and visualizing nanoparticle enrichment [13]. Jin et al. designed a fluorescent ferrimagnetic ferritin nanoplatform (MFtn-Ce6) and achieved very effective imaging results in mouse tumor MRI and FLI imaging [14]. Seraj et al. has formed a new type of targeted nanoparticles by conjugating magnetic particles with anti-mucin 16 (MUC16) adaptors for targeted drug delivery as well as non-invasive MRI contrast agents [15]. Tarighatnia et al. has prepared the Bi-DOPA-PEG-I-MUC16 NPs, and these results have suggested that the MUC-16 aptamer targeted NPs regard as promising targeted CT contrast agents for molecular imaging [16]. However, so far, there have not been many reports on the successful application of fluorescence imaging and MRI in monitoring gastric cancer metastasis. Hence, it is very crucial to establish a method based on MRI/FLI to accurately monitor gastric cancer metastasis in vivo. In this report, we constructed a hollow $\text {Mn}_{3}\text {O}_{4}$ nanocomposites for MRI/FLI dual-modal imaging to improve the imaging accuracy of in situ gastric cancer and metastases. $\text {Mn}_{3}\text {O}_{4}$ was wrapped with TEOS and bMSN, respectively, and then internally etched with an alkaline solution to form $\text {Mn}_{3}\text {O}_{4}$@HMSN (MH NPs) with a hollow coreshell structure. MH NPs was then amino-modified, and cross-linked with Cy7.5 and folic acid on the surface to obtain $\text {Mn}_{3}\text {O}_{4}$@HMSN-Cy7.5-FA (MHCF NPs). After the preparation, the cross-linking of Cy7.5 was verified through UV and fluorescence spectrum results. The tumor microenvironment responsiveness of the nanocomposite was verified by the in vitro MRI imaging and relaxation rate analysis under acidic conditions. The tumor targeting effect of the nanocomposite was further tested through cellular endocytosis and cell affinity experiments. Finally, the accuracy of the nanocomposite in monitoring gastric cancer metastasis in vivo was further explored by in vivo MRI/FLI dual-modal imaging in a gastric cancer orthotopic metastasis model (Fig. 1). Ultimately, we provided a potential nano-diagnostic platform for precise clinical monitoring of gastric cancer metastasis.

## Results

### Synthesis and characterization of MHCF NPs

**Fig. 2:**
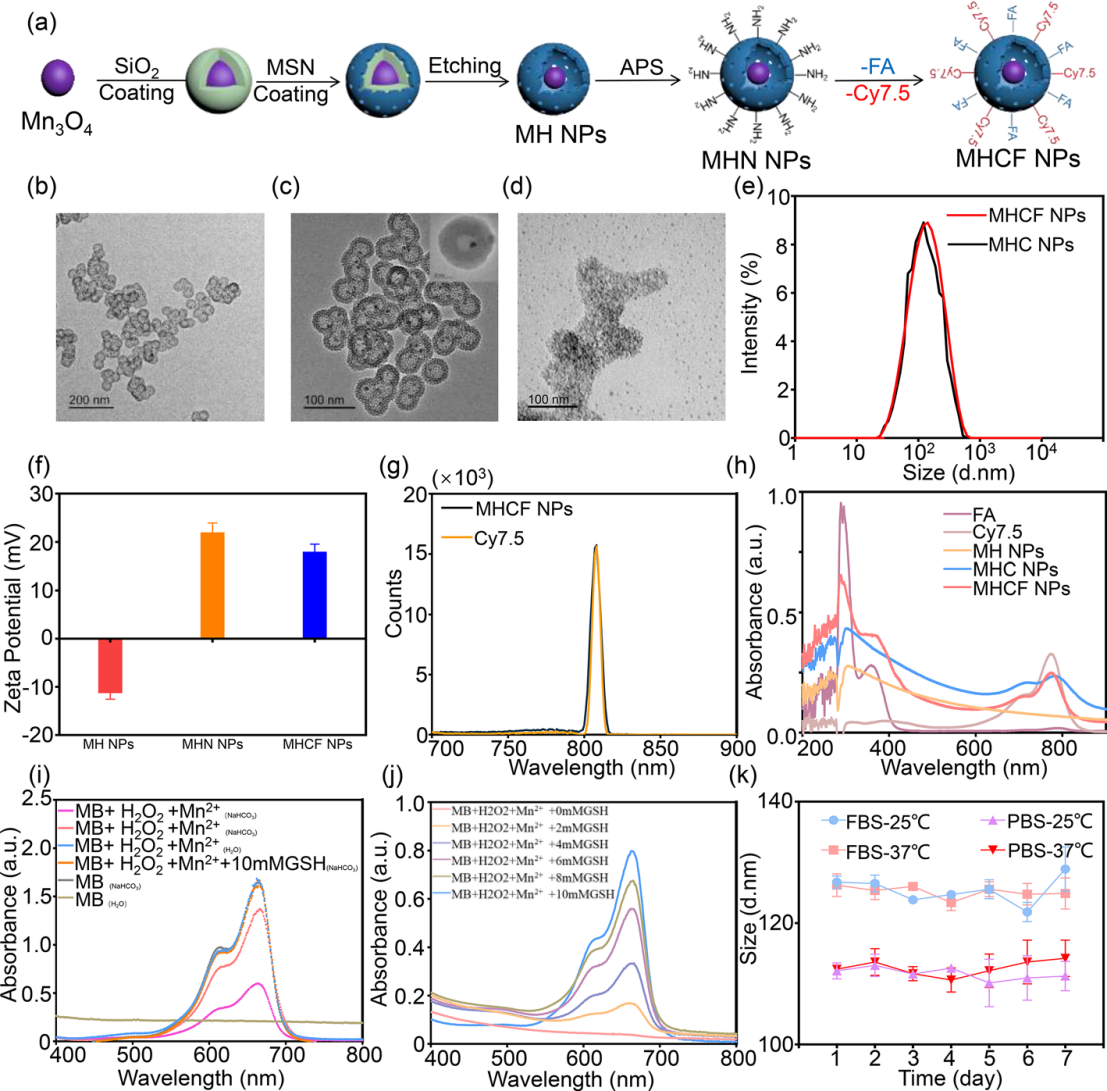
The characterization results of MHCF NPs. **a** Synthesis of MHCF NPs. **b** TEM image of MHC NPs. **c** TEM image of MHCF NPs. **d** TEM image of the degradation of MHCF NPs in lactated Ringer’s solution (LR) at 37 $^{\circ }\hbox {C}$ with slight shaking after 66 h. **e** Hydrated particle size distribution of MHCF NPs. **f** Zeta potential of MHCF NPs, MHN NPs, and MH NPs. **g** Fluorescence spectra of MHCF NPs and Cy7.5. **h** UV–vis absorption spectra of MHCF NPs, MHC NPs, MH NPs, Cy7.5, and FA. **i** UV–vis absorption spectra of ${\text {Mn}^{2+}}$-induced MB degradation in solutions with different components and (**j**) ${\text {Mn}^{2+}}$-induced MB degradation when GSH concentration gradually increased. **k** Hydrated particle size distribution changes of MHCF NPs in different solvents at different temperatures

In order to accurately monitor in situ gastric cancer and metastatic lesions, we constructed MHCF NPs. The water–oil two-phase method was used to wrap and etch the $\text {Mn}_{3}\text {O}_{4}$ surface to form a HMSN layer. The surface was modified with amino groups and cross-linked with Cy7.5 and FA to prepare the nano-silicon composite MHCF NPs (Fig. 2a). $\text {Mn}_{3}\text {O}_{4}$ nanospheres and MHCF NPs were observed by TEM. $\text {Mn}_{3}\text {O}_{4}$ nanospheres are well dispersed, with a size distribution of approximately 10 nm. MHCF NPs has an obvious spherical structure and a uniformly sized hollow mesoporous structure with a size distribution of 54.94 ± 1.62 nm, and the $\text {Mn}_{3}\text {O}_{4}$ nanospheres wrapped in the center are clearly visible (Fig. 2b). The degradation process of MHCF NPs after 66 h was recorded in Fig. 2. At first, the structure of MHCF NPs was intact, and the images clearly showed a core–shell structure. After 66 h, the structure of MHCF NPs had completely degraded, making it unidentifiable in morphology, with a further reduction in size. This process also highlights the excellent in vivo metabolic capability of MHCF NPs (Fig. 2c, d) [15]. The hydrated particle size of MHCF NPs measured by DLS is approximately 113.17 ± 0.90 nm (Fig. 2e), which has a discrepancy with the actual size shown by TEM. This may be caused by the hydration layer on the surface of the nanocomposites. After amino modification of MH NPs, the zeta potential changed from $-$ 10.8 mV to 21.8 mV, confirming the successful modification of the surface amino group (Fig. 2f). The UV–visible light absorption spectra of MH NPs, $\text {Mn}_{3}\text {O}_{4}$@HMSN-Cy7.5 (MHC NPs), MHCF NPs, Cy7.5, and FA were measured to verify whether Cy7.5 was successfully cross-linked. It can be seen that the maximum absorption wavelength of Cy7.5 is located at 796 nm. While the maximum absorption wavelength of MHCF NPs has a red shift of about 15 nm, which may be due to the interaction between MH NPs and Cy7.5. In addition, the red shift of the wavelength is more conducive to subsequent in vivo fluorescence imaging. It also shows that Cy7.5 is successfully cross-linked to the surface of $\text {Mn}_{3}\text {O}_{4}$@HMSN-NH2 (MHN NPs). Based on this, we need to obtain the fluorescence spectra of Cy7.5 and MHCF NPs to determine whether cross-linking affects the fluorescent properties of Cy7.5 (Fig. 2g). The results show that cross-linking Cy7.5 on the surface of the nano-silicon composite did not affect the fluorescence properties of the dye, ensuring that Cy7.5 can exert its fluorescence properties in cell experiments and in vivo imaging experiments. The increase in the UV absorption peak at 790 nm and 285 nm further confirmed the successful cross-linking of Cy7.5 and FA (Fig. 2h). Meanwhile, ${\text {Mn}^{2+}}$ in MHCF NPs can catalyze the generation of ROS by peroxide in acidic conditions. Then, the ability of MHCF NPs at different concentrations of GSH (0, 4, 6, 8, 10 mM) to degrade methylene blue was evaluated. With the concentration of GSH decreasing, the absorbance at 665 nm of each group continued to decrease, and the overall solution color became lighter after the reaction, indicating that MHCF NPs could generate sufficient hydroxyl radicals through a Fenton-like reaction with ${\text {H}_{2}\text {O}_{2}}$ to degrade methylene blue. In addition, the addition of different concentrations of GSH resulted in a deepening of the solution color, suggesting that the generated hydroxyl radicals were cleared by glutathione (Fig. 2i, j). $\text {Mn}_{3}\text {O}_{4}$ served as a manganese-based contrast agent. We evaluated the imaging effect and relaxation rate of nanocomposites under different concentrations and pH conditions. Under the same pH conditions, as the concentration of MHCF NPs increased, the MRI signal also gradually amplified.

**Fig. 3:**
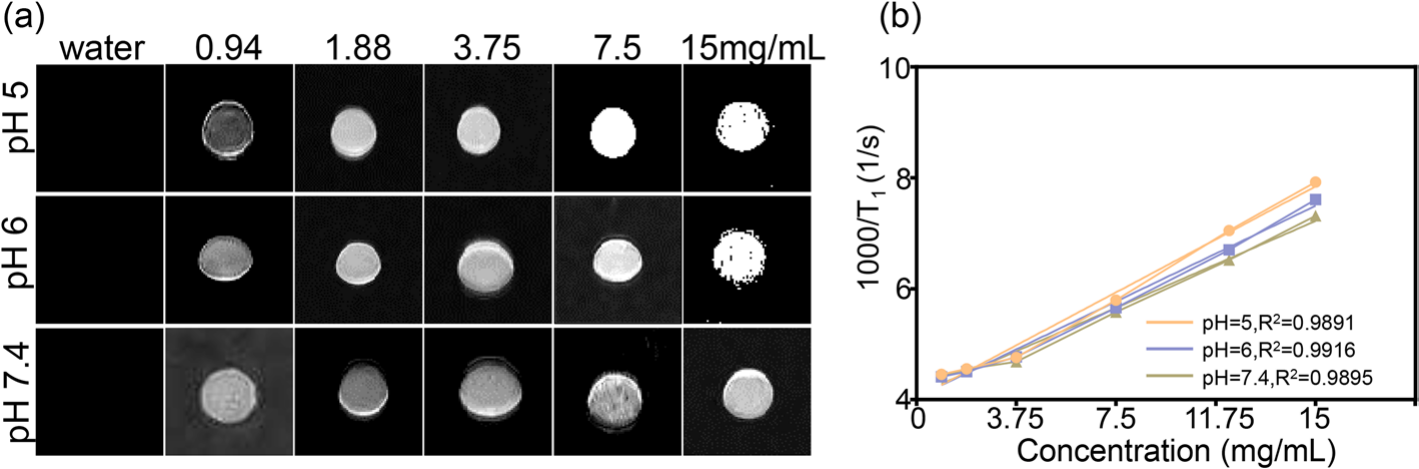
In vitro MRI of MHCF NPs. **a** MRI of MHCF NPs at different concentrations and pH values. **b** MRI relaxation rates of MHCF NPs at different concentrations and pH values

With the same concentration, MHCF NPs under acidic environment showed stronger MRI signals than those of the other environments, preliminarily showing that MHCF NPs can respond to the acidic microenvironment (Fig. 3a). The ${\text {T}_{1}}$ relaxation time of MHCF NPs nano-silicon composites at different concentrations was measured at pH = 7.4, 6, and 5. The results show that as the pH dropped, the relaxation rate gradually rose. When the pH was 5, the relaxation rate was 0.085, which is 1.4 times the relaxation rate of pH 7.4, indicating that MHCF NPs had better MRI effects in tumor acidic microenvironments (Fig. 3b). Under acidic conditions, $\text {Mn}_{3}\text {O}_{4}$ will accelerate the release of ${\text {Mn}^{2+}}$, thereby increasing the relaxation rate and presenting better imaging effects. The stability of nanoparticles is an important factor in long-term imaging studies in vivo. MHCF NPs was dispersed in phosphate buffer saline (PBS) and 10 % fetal bovine serum (FBS), and the changes in particle size at different temperatures were measured to verify the stability of the nanocomposites. The particle size distribution of MHCF NPs remained relatively stable under different conditions (Fig. 2k). The results show that MHCF NPs have good stability and can be used in subsequent in vivo experiments.

### In vitro and in vivo biocompatibility of MHCF NPs

**Fig. 4:**
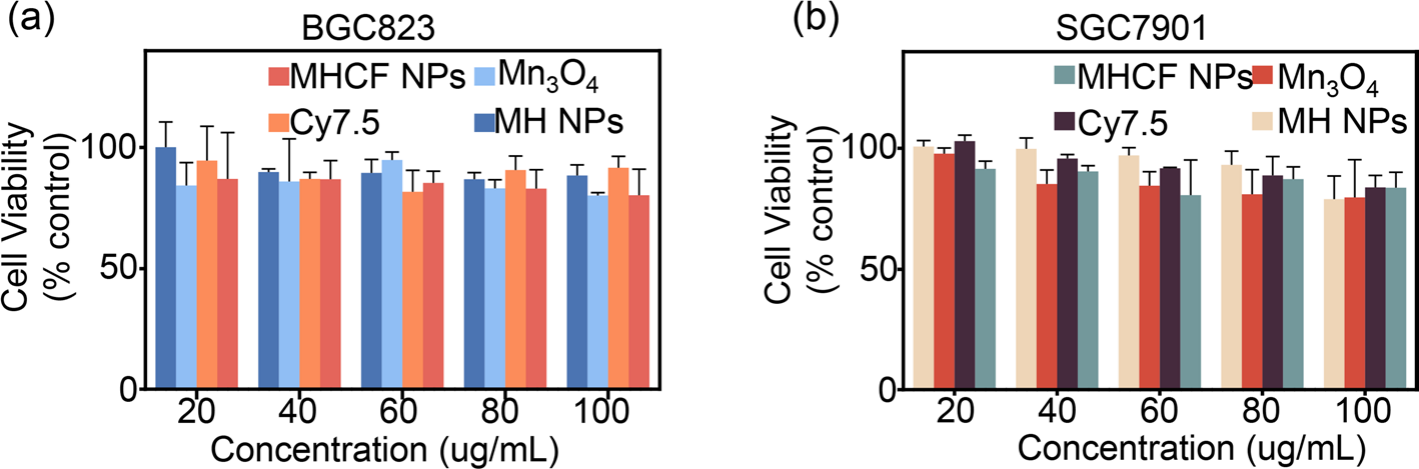
The inhibitory effects of MHCF NPs on tumor cells. **a** Survival rate of BGC823 cells incubated with MHCF NPs. **b** Survival rate of SGC7901 cells incubated with MHCF NPs

**Fig. 5:**
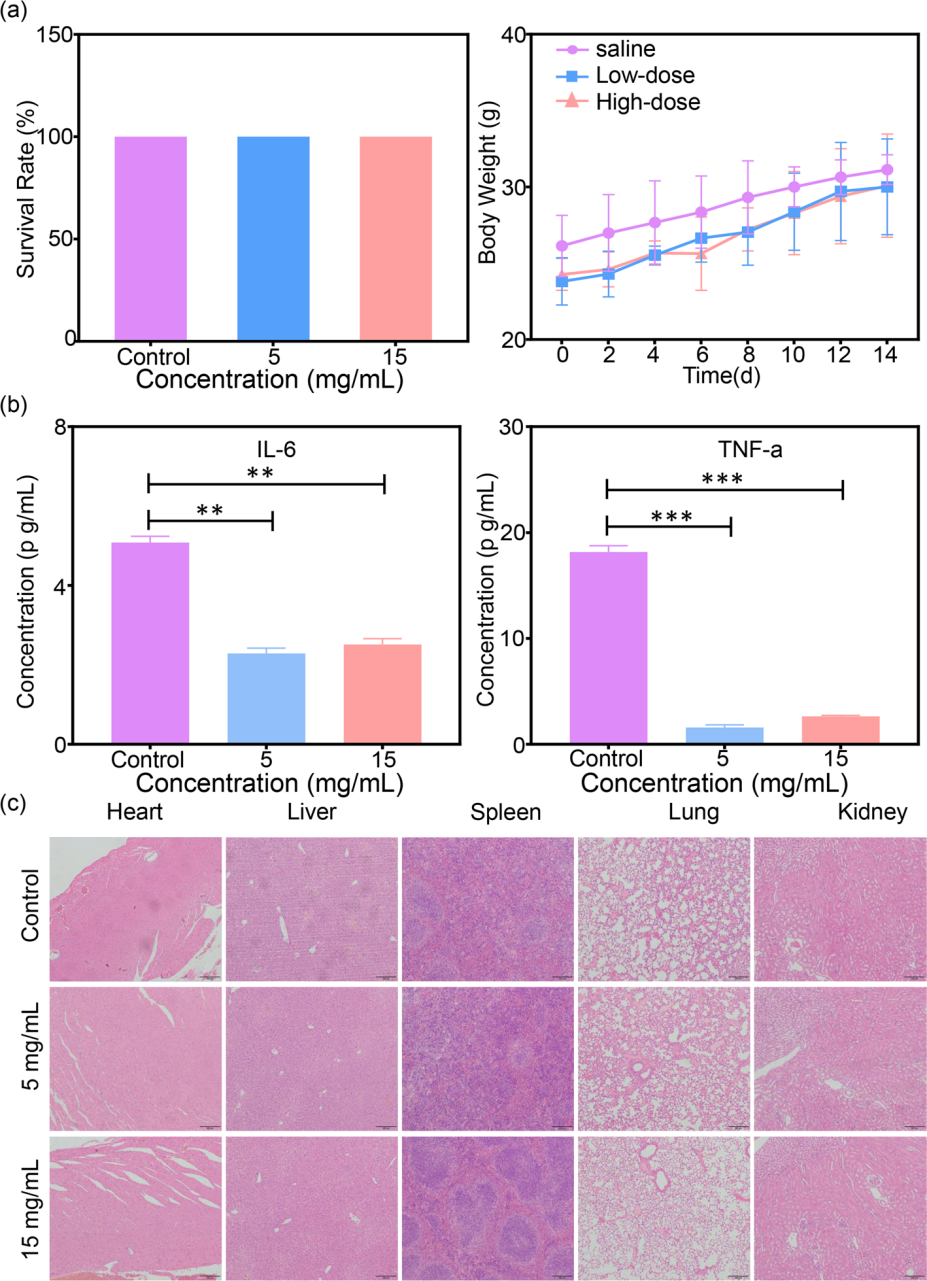
The biocompatibility of MHCF NPs in vitro and vivo. **a** Survival rate and body weight of the mice. **b** Blood biochemistry results of IL-6 and TNF-a. **c** H&E staining of mouse major organ tissues (heart, liver, spleen, lung, and kidney), scale bar = $200\,\upmu \hbox {m}$

**Fig. 6:**
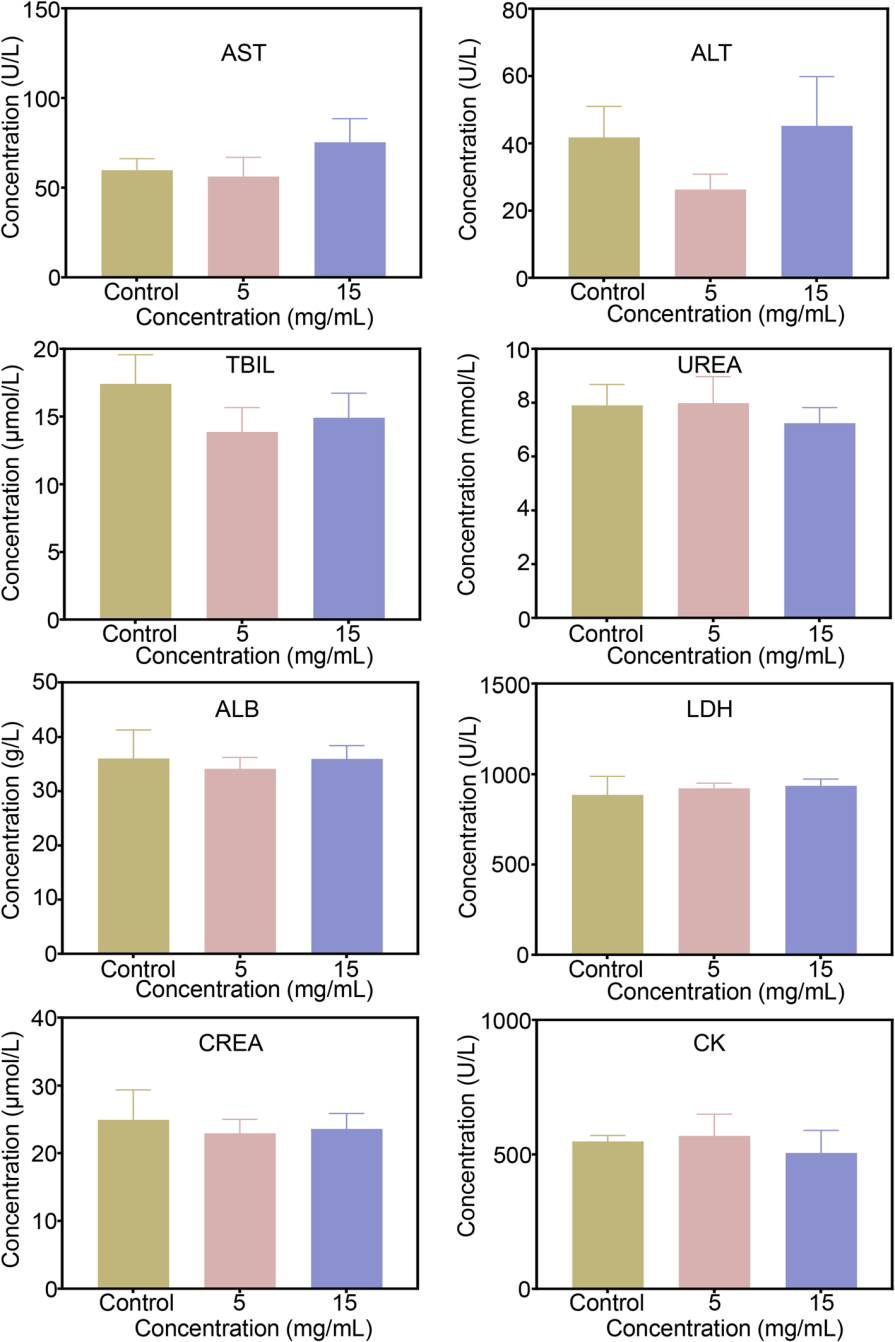
The blood biochemical results of AST, ALT, TBIL, UREA, ALB, LDH, CREA, and CK

The biosafety of nanocomposites is crucial for subsequent in vivo applications. In order to verify the biosafety of MHCF NPs, SGC7901 and BGC823 cells were used as experimental subjects, and the MTT method was performed to test cytotoxicity. In SGC7901 and BGC823 cells, as the concentration of MHCF NPs increased, the cell survival rate did not decrease significantly, indicating that MHCF NPs has a minor the toxic side effects of on cells and a good biocompatibility, and can be used for subsequent in vivo and in vitro experiments (Fig. 4a, b). To further verify that MHCF NPs can be used for in vivo studies, mice were injected with MHCF NPs in 5 mg mL$^{-1}$ and 15 mg mL$^{-1}$ , respectively. The weight changes of the mice were observed within 14 days. The results show that all 30 mice survived, and the weight of mice in each group shows a steady upward trend. After the mice were euthanized, hematoxylin and eosin staining (H&E staining) was performed on the heart, liver, kidney, spleen, and lungs. High and low concentrations of MHCF NPs show no obvious damage to the organs. H &E sections show that the cell morphology of each organ tissue was almost consistent with that of the control group (Fig. 5). By extracting the serum of mice, the blood biochemical indicators of mice were tested. AST, ALT, TBIL, UREA, ALB, LDH, CREA, and CK indicators were all within the normal range. However, compared with the control group, the levels of IL-6 and TNF-a were significantly reduced. The above results show that the MHCF NPs has good biosafety (Fig. 6).

**Fig. 7:**
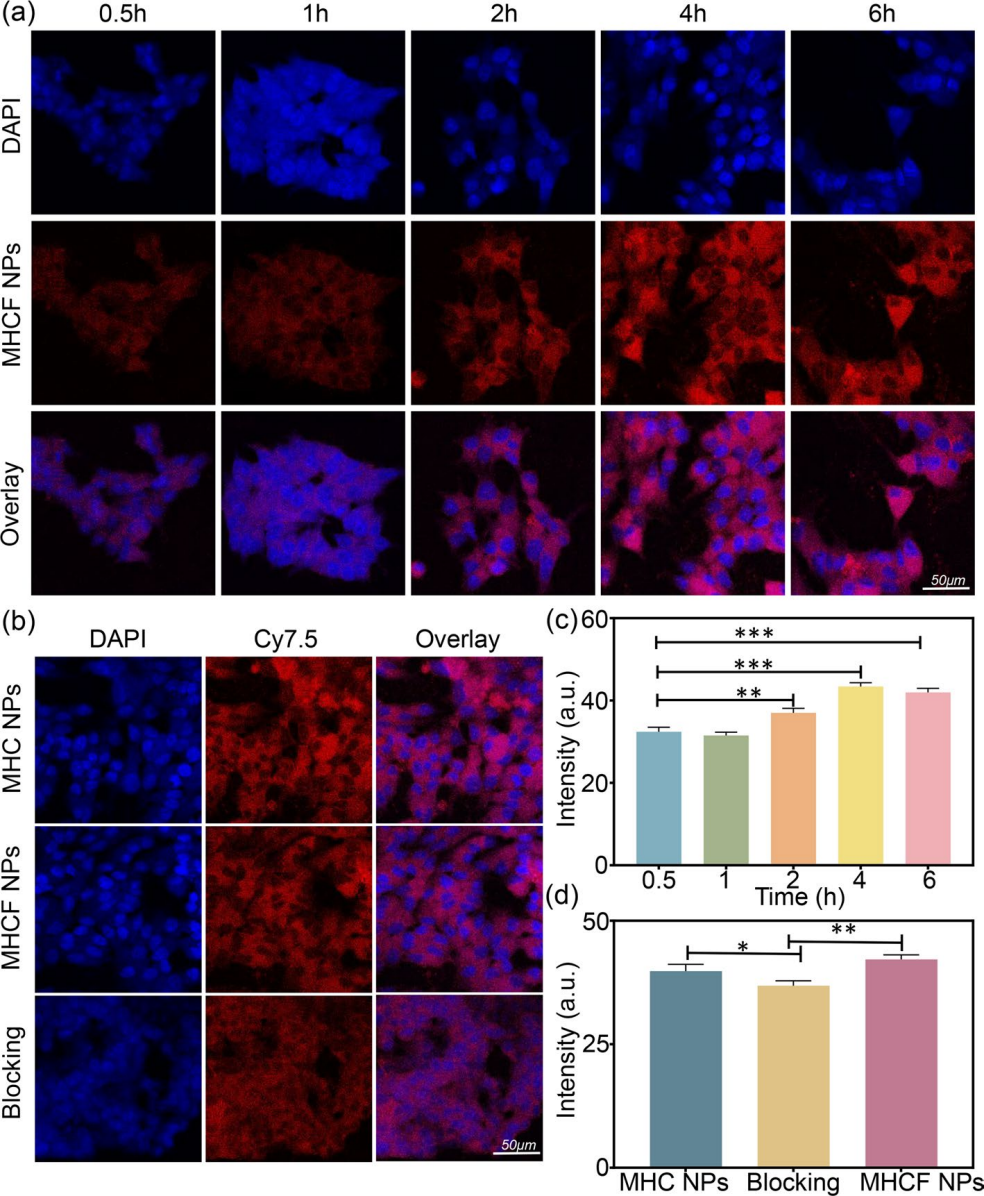
Cellular uptake and affinity analysis of MHCF NPs. **a** CLSM images of cellular endocytosis of MHCF NPs at different time points, scale bar = 50 $\upmu$m. **b** CLSM images of cellular affinity of MHCF NPs, scale bar = 50 $\upmu$m. **c** Quantitative results of fluorescence intensity of cellular endocytosis of MHCF NPs. **d** Quantitative results of fluorescence intensity of cellular affinity of MHCF NPs

### Cellular uptake and affinity analysis of MHCF NPs

Nanocomposites have a targeting effect that can not only be specifically recognized by tumor cells, but also have an enrichment effect. We realized targeting effects on MHCF NPs by cross-linking folic acid on the surface of MHC NPs. Folic acid (FA) can be recognized by specific proteins on the surface of tumor cells, thereby targeting nanocomposites to the tumor site. We used cellular endocytosis experiments to test the endocytic behavior of MHCF NPs and its targeting effect to tumor cells. Firstly, the same concentration of MHCF NPs was co-incubated with SGC7901 cells, and then the fluorescence intensity was observed with a confocal microscope. Blue fluorescence was from diamidino-phenyl-indole (DAPI), and red fluorescence was from MHCF NPs. As the incubation time extended, the red fluorescence in the cells gradually increased and was mainly distributed in the cytoplasm (Fig. 7a). Through quantitative analysis of fluorescence intensity (Fig. 7c), we found that the endocytosis of nanocomposites by cells reached the maximum at 4 h, and the fluorescence intensity after 2 h of incubation was significantly different from that at 0.5 h, which is consistent with the cell staining image results ($${^{**} {P}} < 0.01$$, $${^{***}{P}} < 0.001$$). Secondly, we verified the cell affinity of MHCF NPs. In the FA-blocked group, since FA bound to the target site on the tumor surface and inhibited the endocytosis of MHCF NPs by cells, the red fluorescence was weaker than that of the targeted group. In the targeted group, MHCF NPs was better uptaken by SGC7901 cells, while the non-targeted group under the same treatments showed slightly lower fluorescence intensity, indicating that the addition of FA enhanced the targeting effect of the nano-silica complex in tumor cells (Fig. 7b, d). Through quantitative analysis of fluorescence intensity, we found that it is consistent with the above cell imaging results ($${^{*}{P}} < 0.05$$, $${^{**}{P}} < 0.01$$).

### In vivo MRI and FLI for orthotopic gastric cancer and lung metastases mice model

**Fig. 8:**
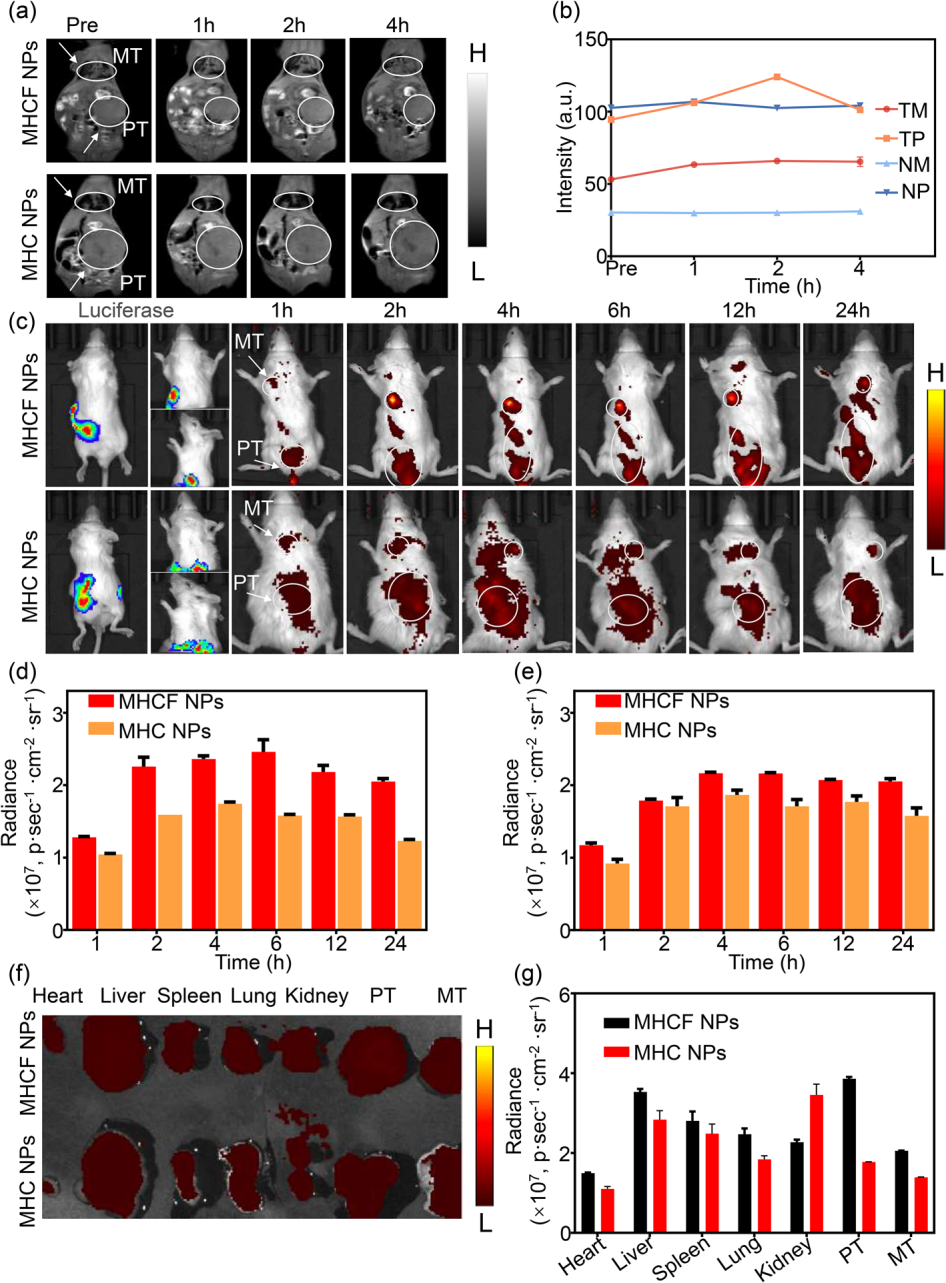
The in vivo tumor MRI, FLI, and bioluminescent imaging (BLI) of MHCF NPs. **a** In vivo MRI of the mice in MHCF NPs and MHC NPs groups at different times. **b** MRI relaxation rates of the mice in each group. **c** In vivo fluorescence and bioluminescence images of the mice in MHCF NPs and MHC NPs groups at different times. **d** Quantitative results of the intensity of fluorescence images of primary tumors. **e** Quantitative results of the intensity of fluorescence images of metastatic tumors. **f** Fluorescence images of tumors and major organs of MHCF NPs. **g** Quantitative results of fluorescence intensity of tumors and major organs of MHCF NPs

The MRI and FLI functions of MHCF NPs were tested by in vitro experiments. We successfully verified that the nanocomposites have excellent imaging and targeting effects in the tumor acidic microenvironment. Since the in vivo environment is more complex than that of the in vitro, we conducted MRI and FLI experiments on in situ gastric cancer and metastatic lesions to further prove its dual-modal imaging function in vivo. Here PT (primary tumor) and MT (metastasis tumor) stand for primary tumor and metastasis tumor, respectively. The results show that the MRI signal was significantly enhanced after the injection of MHCF NPs, and the lung signal in the targeting group was also significantly enhanced. This result also proves the successful construction of lung metastases. In the signal intensity quantitative results, we found that MHCF NPs’ tumor cell targeting effects were better than the that of the control group (nanocomposites without FA). Due to the targeting effect of FA, the MRI signal is significantly enhanced in not only the original tumor, but also the metastatic tumor in lungs (Fig. 8a, b). However, MRI images show that the intestine also had a certain amount of signal, which may be caused by the remaining high-fat mouse food in the model mice. Therefore, in subsequent experiments, we further conducted imaging experiments on in situ gastric cancer and metastatic lesions through NIR-I FLI. This result confirms that MHCF NPs has the potential to accurately detect in situ tumors and their metastases using MRI. Moreover, the MRI signal rose and drop from 0 to 4 h. During this period, the nano-silica nanocomposites accumulated in the tumor site but then were excreted by the body metabolism, thus causing a decrease in MRI signal intensity. This scenario indicates that the nanocomposites have a short metabolism time and have little toxic side effects in the body. In order to further verify the accuracy of MRI, we performed fluorescence positioning through the targeting effect of nanocomposites. Setting up MHC NPs and MHCF NPs groups, the bioluminescence image clearly showed the location of gastric cancer in situ tumors and metastases. As the time after injection extending, the fluorescence intensity of the tumor site in the model mice gradually increased, and the tumor location can be accurately located in the active targeting group, indicating that the MHCF NPs has an excellent targeting effect on gastric cancer tumor cells (Fig. 8c). The fluorescence signals of gastric cancer in situ tumors and metastases of the two groups of model mice were quantitatively analyzed (Fig. 8d). After injection, the fluorescence signal of the in situ tumors of the model mice in the targeted group gradually increased over time. The signal reached a relatively high level after 2 h and started decreasing after 6 h. However, the signal of MHC NPs group was significantly lower than that of MHCF NPs group, and had no significant change. The fluorescence signal of metastases in MHCF NPs group was also significantly enhanced 2 h after injection, while the signal in MHC NPs group did not change much either (Fig. 8e). Quantitative results were consistent with in vivo imaging results. Since magnetic particles imaging is faster than fluorescent imaging, the maximum intensity of MRI imaging appeared 2 h after injection, while for fluorescence imaging the maximum intensity was at 6 h. The above results show that the MHCF NPs has good targeting ability to gastric cancer tumor cells, and can effectively monitor gastric cancer metastases through in vivo MRI and FLI. After the in vivo FLI was completed, the model mice were euthanized and the main organs and tumor were retained for fluorescence imaging. All organs in the targeted group had fluorescence signals. The signal at the tumor site of MHCF NPs was significantly stronger than that of MHC NPs (Fig. 8f). The fluorescence signals of the organs and tumors of were quantitatively analyzed (Fig. 8g). Except for the kidney and tumor sites, the difference in fluorescence intensity between the targeted and non-targeted groups was not significant for other organs, which was consistent with the in vivo imaging results. These results further verify the excellent tumor targeting effects of MHCF NPs. We can realize in vivo monitoring of tumor metastasis through FLI with MHCF NPs. The FLI can further verify the accuracy of MRI imaging in in situ gastric cancer to achieve precise diagnosis.

## Methods

### Materials

The human gastric cancer cell line SGC 7901 (sourced from ATCC) was purchased from Procell Life Science and Technology Co., Ltd. (Wuhan, China). Reduced glutathione (GSH), manganese acetate, oleic acid, oleylamine, dopamine hydrochloride, methylene blue (MB), and 1-(3-dimethylaminopropyl)-3-ethyl were purchased from Sigma-Aldrich. Carbodiimide hydrochloride (EDC), fluorescein diacetate (FDA), and paraformaldehyde (4$\%$) were purchased from Aladdin. 4,6-Diamidino-2-phenylindole (DAPI) was purchased from Boster Biological Technology. Propidium iodide (PI) was purchased from Beyotime Biotechnology.

### Synthesis of MHCF NPs

Manganese acetate (1 mmoL, 0.17 g), oleic acid (640 $\upmu$L), and oleylamine (3.28 mL) were dissolved and mixed in xylene (15 mL). The mixture solution was then slowly heated to $90\,^{\circ }\hbox {C}$ and reacted for 10 min. Deionized water (1 mL) was then added to the solution, and the solution continued reacting at $90\,^{\circ }\hbox {C}$ for 2.5 h. Then, absolute ethanol was added and centrifuged. The precipitate was dispersed in cyclohexane to obtain $\text {Mn}_{3}\text {O}_{4}$ solution (solution 1). Meanwhile, IGE-PAL CO-520 (2 mL) was dispersed with cyclohexane (40 mL) and stirred for 40 min (solution 2). Subsequently, solution 1 (0.5 mL) was added to the solution 2 under stirring at room temperature for 2 h (solution 3). Finally, ammonia water (0.28 mL) was added to the solution 3 and stirred it for 2 h (solution 4). Then, TEOS (0.5 mL) was slowly added to the solution 4 and reacted for 48 h before methanol being added to terminate the reaction (solution 5). Afterward, solution 5 was centrifuged and the precipitate was dispersed in 10 mL of deionized water. In addition, 10 $\%$ triethanolamine aqueous solution (3 mL) were mixed with CTAC aqueous solution 20 mL, and reacted at room temperature for 1.5 h, then added the above solution 5 to react at room temperature for 1.5 h. The reaction was then moved to an $80\,^{\circ }\hbox {C}$ water bath and added with TEOS (300 $\upmu$L) to react for another 1 h (solution 6) [18, 19]. Solution 6 was moved to a $50^{\circ }\hbox {C}$ water bath for 1 h, and then centrifuged quickly to obtain the precipitate. The precipitate was then washed with sodium chloride–methanol solution and finally dispersed into absolute ethanol (solution 7). Solution 7 was added with 1 mL of APS and reacted at 85–$90^{\circ }\hbox {C}$ for 48 h. The solution was then centrifuged and the precipitate was dispersed into deionized water to obtain MHN solution (solution 8). Subsequently, Cy7.5 (5 $\upmu$L) was added to the solution 8 and stirred in a centrifuge tube for 2 h. Afterwards, centrifuged the solution and washed the precipitate with water to obtain the MHC aqueous solution (solution 9). Meanwhile, FA, EDC, and NHS (0.0047 g FA, 0.002 g EDC and 0.0062 g NHS) were dispersed with 500 $\upmu$L DMSO and activated for 30 min. Solution 9 was then added to the activated solution, stirred for 3 h, and finally dispersed in deionized water to obtain MHCF NPs [20, 21].

### Characterization of MHCF NPs

The morphology of MHCF NPs was observed by TEM. The particle size distribution and zeta potential of MHCF NPs were measured using Nano ZS90 zeta-sizer. We used a UV–visible spectrophotometer to capture the absorption spectra of MH NPs, MHC NPs, MHCF NPs, FA, and Cy7.5 under the wavelength between 200–1000 nm. The fluorescence spectra of MHCF NPs and Cy7.5 were measured with a fluorescence spectrometer (excitation wavelength: 790 nm). MHCF NPs’ stability in different environments in vitro was evaluated by measuring the particle size distribution changes in different solutions. MHCF NPs were placed in PBS $25\,^{\circ }\hbox {C}$, PBS $37\,^{\circ }\hbox {C}$, FBS $25\,^{\circ }\hbox {C}$ and FBS $37\,^{\circ }\hbox {C}$ and the size distributions were measured at multiple time points.

### In vitro MR imaging of MHCF NPs

The method was as follows: we diluted MHCF NPs into 5 concentration gradients (0.94 mg/mL, 1.88 mg/mL, 3.75 mg/mL, 7.5 mg/mL, and 15 mg/mL) and placed it in a 200-mL centrifuge tube to obtain the ${\text {T}_{1}}$ relaxation times corresponding to different concentrations. Also, MHCF NPs solutions with different pH (7.4, 6, and 5) at corresponding concentrations were prepared. The parameter settings of the conventional spin echo acquisition sequence were as follows: TR = 400 ms, TE = 18.2 ms, slice gap = 0.8 mm, slice width = 3 mm. The value of r1 was calculated by the linear regression of 1/${\text {T}_{1}}$(S1) and the concentration (mm) of MHCF NPs. In order to verify the ${\text {T}_{1}}$-weighted MRI properties of MHCF NPs, a 0.5T small animal MRI scanner was used to obtain the relaxation images and relaxation times. Deionized water were used as control group, and the relaxation images and relaxation times of MHCF NPs at different concentrations (0.94 mg/mL, 1.88 mg/mL, 3.75 mg/mL, 7.5 mg/mL, and 15 mg/mL) and different pH values (pH 7.4, pH 6, pH 5) were measured for us to create the scatterplot of the relaxation rate vs relaxation time.

### In vitro cell viability of MHCF NPs

SGC7901 and BGC823 cell suspensions were added to a 96-well plate at 100 $\upmu$L/well ($10^{5}$ cells/mL) and incubate for 24 h ($37\,^{\circ }\hbox {C}$, 5$\%$ $\text {CO}_2$). MHCF NPs, $\text {Mn}_{3}\text {O}_{4}$, MH, and MHC with different concentrations (20 $\upmu$g/mL, 40 $\upmu$g/mL, 60 $\upmu$g/mL, 80 $\upmu$g/mL, and 100 $\upmu$g/mL) were added to the plate and incubated for 24 h. We then removed the medium, washed the cells 3 times with PBS, and added new medium. 10 $\upmu$L of MTT solution was added to each well. After 2 h of incubation, the absorbance at 490 nm was measured with a microplate reader. Finally, we calculated the cell viability according to the following formula (OD is the optical density value, $\text {OD}_{\text {black}}$ is the absorbance value of the blank unspiked cell group, $\text {OD}_{\text {MHCFNPs}}$ is the absorbance value of the treated cells, $\text {OD}_{\text {control}}$ is the absorbance value of the untreated cells):

1 $$\begin{aligned} {\text{Cell viability}}\, ( \%)=[( \text {OD}_{\text {MHCFNPs}}- \text {OD}_{\text {black}})/( \text {OD}_{\text {control}}- \text {OD}_{\text {black}})]. \end{aligned}$$

### Cellular uptake and affinity analysis of MHCF NPs

Suspended SGC7901 cells were added to a confocal dishes and culture at $37\,^{\circ }\hbox {C}$ with 5$\%$ $\text {CO}_2$. When the cells adhered to the wall and grew to 50–60$\%$, MHCF NPs were added to the dishes to be incubated for 1 h, 3 h, 6 h, and 9 h, respectively. When the time is up, we removed the culture medium and washed the cells with PBS. Then, we quickly added 4$\%$ paraformaldehyde and waited for 20 min to fix the cells. DAPI dye was added for staining. We observed the cells with a laser confocal microscope, and tested the affinity of the cells by the same method. We added SGC7901 cells suspension to a confocal petri dish, and after the cells had adhered to the wall, FA was added to bind to the tumor surface-specific receptor. After the cells were treated with FA for 2 h, the old medium was replaced with fresh one, and MHCF NPs was added to the dish. After blocking, the old medium was replaced with fresh one, and MHCF NPs was added to the dish. The dish was then incubated for 4 h, fixed with 4$\%$ paraformaldehyde, washed with PBS, stained with DAPI dye, and observed with a laser confocal microscope. Quantitative analysis of all confocal images was carried out with Image J software.

### Orthotopic gastric cancer and lung metastases mice model

All cells were cultured in 10$\%$ FBS medium and incubated at $37\,^{\circ }\hbox {C}$ with 5$\%$ $\text {CO}_2$. Severe combined immunodeficiency (SCID) mice (4 weeks old, approximately 16 g) were purchased from Chongqing Tengxin Biotechnology Co., Ltd. Mice were fed under SPF conditions for 1 week. Orthotopic gastric cancer and lung metastasis mouse models were used for in vivo MRI and near-infrared first-zone fluorescence imaging. To build the model, 100 $\upmu$L of SGC7901-Luc cells (5$\times$ $10^6$ cells/mL) were injected into the stomach wall of mice during a surgery. After injection, the mice were fed under SPF conditions. The tumor size was monitored by bioluminescence imaging. The animal study protocol was approved by accordance with the animal ethics of the affiliated institutions (No. KYLL-2021-609) [17]Chongqing Tengxin Biotechnology Co., Ltd.

### In vivo MRI and FLI for orthotopic gastric cancer and lung metastases

MHC NPs and MHCF NPs were injected intravenously into two groups of tumor-bearing mice, respectively. The control group was set up stroke-physiological saline solution. ${\text {T}_{1}}$-weighted MRI images of gastric cancer metastasis model mice were obtained at different time points. MHC NPs (5 mg/mL, 200 $\upmu$L), and MHCF NPs (5 mg/mL, 200 $\upmu$L) were injected intravenously to the two groups of tumor-bearing mice, respectively, the control group was set up stroke-physiological saline solution. The IVIS system was used to obtain fluorescence images of gastric cancer metastasis model mice in each group at different time points. After imaging, the model mice were euthanized, and the heart, liver, spleen, lung, kidney, and tumor were obtained. The fluorescence distribution of the materials in the tumor and organs was analyzed [22, 23].

### In vivo acute toxicity

15 female and 15 male healthy mice were randomly divided into three groups, each group containing 5 female and 5 male mice. The mice in the first, second, and third group were injected with 200 $\upmu$L PBS, 200 $\upmu$L MHCF NPs (5 mg mL$^{-1}$), and 200 $\upmu$L MHCF NPs (15 mg mL$^{-1}$), respectively. The weight and number of death of mice in each group within 14 days were recorded. The survival rate of mice was calculated. After 14 days, the mice were euthanized, and the main organs of the mice were retained for H&E staining.

### Statistical analysis

SPSS and Graphpad prism 5.0 were used for statistical analysis. Multiple components were analyzed using single factor variance analysis. Tests between two groups were performed with t-test. There was a significant difference between the groups when (${^{*}{P}} < 0.05$).

## Conclusion

In this paper, we used TEOS and bMSN to encapsulate $\text {Mn}_{3}\text {O}_{4}$ and performed surface etching with alkaline solution to synthesize a hollow core–shell structured MH NPs. Fluorescent dye and FA were cross-linked to the surface of MH NPs to construct a new type of MRI/FLI dual-modal imaging guided hollow core–shell structure nanocomposite MHCF NPs. In vitro characterization results show that the MHCF NPs was successfully synthesized and had a size distribution of approximately 100 nm. UV and fluorescence spectrum tests verify that the nanocomposite had an obvious absorption peak at 808 nm, indicating a successful cross-linking of Cy7.5. Stability tests were conducted in different media at different temperatures. The nanocomposites can be stably dispersed in different physiological environments for a fairly long time. In vitro MRI imaging and relaxation rate analysis show that the nanocomposite had good response performance to the acidic microenvironment of the tumor, and the relaxation rate of $\text {Mn}_{3}\text {O}_{4}$ was significantly increased at pH = 5. Cellular endocytosis and affinity experiments further demonstrate that the surface FA enhanced the targeting effect of MHCF NPs to tumor cells. Through cytotoxicity and in vivo acute toxicity tests, we found that the nanocomposites have good biological safety. In vivo MRI/FLI imaging results show that the nanocomposites can achieve accurate monitoring of gastric cancer tumors in situ and metastases. BLI’s tumor location information further supports the good accuracy of MRI/FLI dual-modality imaging. The above results show that the MHCF NPs can serve as a good nano-diagnostic platform for precise in vivo monitoring of tumor metastasis. This nanocomposite provides more possibilities for the diagnosis and therapy of gastric cancer metastases.

Editorial Policies for:

Springer journals and proceedings: https://www.springer.com/gp/editorial-policies

Nature Portfolio journals: https://www.nature.com/nature-research/editorial-policies

*Scientific Reports*: https://www.nature.com/srep/journal-policies/editorial-policies

BMC journals: https://www.biomedcentral.com/getpublished/editorial-policies

## Data Availability

The data are contained within the article.

## Materials availability

The materials are contained within the article.
